# Facilitated Adaptation as A Conservation Tool in the Present Climate Change Context: A Methodological Guide

**DOI:** 10.3390/plants12061258

**Published:** 2023-03-10

**Authors:** Elena Torres, Alfredo García-Fernández, Diana Iñigo, Carlos Lara-Romero, Javier Morente-López, Samuel Prieto-Benítez, María Luisa Rubio Teso, José M. Iriondo

**Affiliations:** 1 Departamento de Biotecnología-Biología Vegetal, Universidad Politécnica de Madrid, 28040 Madrid, Spain; 2 Grupo de Ecología Evolutiva (ECOEVO), Área de Biodiversidad y Conservación, Departamento de Biología, Geología, Física y Química Inorgánica, Universidad Rey Juan Carlos, 28933 Móstoles, Spain; 3 Grupo de Investigación de Ecología y Evolución en Islas, Instituto de Productos Naturales y Agrobiología (IPNA-CSIC), 38206 Tenerife, Spain; 4 Ecotoxicology of Air Pollution, Environmental Department, CIEMAT, 28040 Madrid, Spain

**Keywords:** assisted evolution, artificial selection, assisted gene flow, climate change, evolutionary adaptation, evolutionary rescue, genetics of adaptation, selective breeding

## Abstract

Climate change poses a novel threat to biodiversity that urgently requires the development of adequate conservation strategies. Living organisms respond to environmental change by migrating to locations where their ecological niche is preserved or by adapting to the new environment. While the first response has been used to develop, discuss and implement the strategy of assisted migration, facilitated adaptation is only beginning to be considered as a potential approach. Here, we present a review of the conceptual framework for facilitated adaptation, integrating advances and methodologies from different disciplines. Briefly, facilitated adaptation involves a population reinforcement that introduces beneficial alleles to enable the evolutionary adaptation of a focal population to pressing environmental conditions. To this purpose, we propose two methodological approaches. The first one (called pre-existing adaptation approach) is based on using pre-adapted genotypes existing in the focal population, in other populations, or even in closely related species. The second approach (called de novo adaptation approach) aims to generate new pre-adapted genotypes from the diversity present in the species through artificial selection. For each approach, we present a stage-by-stage procedure, with some techniques that can be used for its implementation. The associated risks and difficulties of each approach are also discussed.

## 1. Introduction

Climate change has turned out to be one of the main threats to biodiversity. The continuous increase in CO_2_ concentration and other greenhouse gases since the industrial revolution is prompting a cascade of environmental changes that are altering the abundance and distribution of many species, carrying some of them towards extinction and disturbing the interactions present in the ecosystems [1,2]. In response to global warming, many plant populations are shifting location to keep themselves within their ecological range [3,4,5,6]. However, this response is not possible for many other species whose dispersal is limited by their own nature [7] or hindered by landscape fragmentation [8,9]. In addition to migration to more favorable locations, plants can also respond to climate change through phenotypic plasticity (i.e., changes in the morphology and physiology that a given genotype experiences in response to different environmental conditions, due to differences in gene expression) and genetic adaptation (i.e., changes in the frequency of adaptive alleles as a result of natural selection that increase fitness) [10,11,12,13]. By contrast, the evolutionary response of many other species is limited by a lack of relevant genetic variability [14,15]. The present speed of global warming is another factor that increases the number of species threatened by climate change [16]. Despite having populations with large genetic diversity, species with low reproductive rates or long generation times may become extinct because their rate of evolutionary adaptation may not be able to match the speed of global warming [17,18,19].

Management actions such as promoting habitat connectivity, establishment of microclimatic refuges or habitat restoration could, therefore, not be enough to warrant the medium and long-term viability of many of these species [9,20]. As a result, two conservation strategies have emerged. The first strategy is known as “assisted migration”, “assisted colonization”, “managed relocation” or “benign introduction”, among others [21], and consists of the intentional movement of individuals to an area that is outside their present distribution range, where climate models forecast that future environmental conditions will be favorable for the target species [22,23]. Since assisted migration was proposed, the debate has centered on the uncertainties derived from the introduction of a new species in an ecosystem [24,25]. There are numerous examples of ecosystems that have been seriously altered as a result of the introduction of a species [26,27], even in the case of threatened species [28,29]. The second strategy, namely, “facilitated adaptation”, is very different because it assumes the change in the environmental conditions of the place where the target species occurs, and it aims to promote the evolutionary adaptation to the new conditions by introducing individuals with alleles that provide beneficial traits (i.e., pre-adapted genotypes) [30,31,32].

The development of “omics” technologies has advanced the knowledge of gene functions and the effect of environment in their expression [33]. Based on these advances, several authors have reviewed, both from theoretical and experimental approaches, the mechanisms of adaptation in the context of climate change, setting the theoretical basis for facilitated adaptation as a conservation strategy [34,35]. In parallel, the advancement in the theory of evolutionary rescue (which relates population dynamics to genetic adaptation) and experimental results on some study cases show that it is possible to intervene in the process [36,37,38]. That is, a population can recover from an environmentally driven decline if a certain number of individuals with adaptive genotypes is supplied at a sufficient rate. Despite the growing body of theoretic evidence to support that facilitated adaptation could work in particular situations, it has not yet been included in management practices and conservation policies. This may be due to the precautionary principle historically applied by conservation biologists focusing on maintaining evolutionary significant units and prioritizing the genetic uniqueness, but also because a methodology on which to base its implementation remains undeveloped.

In this article we review the concept of facilitated adaptation and present two different methodological approaches that may be used to promote the process of genetic adaptation of populations to climate change. We also discuss the risks that may derive from the introduction of pre-adapted individuals and the limitations of these approaches. We present some techniques that could be implemented to carry out the different steps, being aware that they will have to be optimized as knowledge on gene function and interactions between different organization levels improve, and better molecular and computational tools are made available. In any case, since this proposal is conceived for a wide spectrum of plants, the steps will necessarily have to be adapted and more precisely specified when applied to a particular case.

## 2. A Brief Overview of the Conceptual Framework

### 2.1. Evolutionary Adaptation

Adaptation is an evolutionary process derived from natural selection that allows species to respond to biotic and abiotic stresses. Mutation, migration and genetic drift also play a role by facilitating or hindering this process [39]. Consequently, to establish the methodological foundations of facilitated adaptation, it is essential to understand how selection operates and the way the rest of the evolutionary forces modulate this process.

One first issue to consider is the genetic architecture of the adaptive traits involved in the response to climate change. In the case of monogenic traits, genetic adaptation can be acquired through mutations that confer higher fitness. The low initial frequency of a new allele may bring it to extinction through genetic drift, regardless of its adaptive value. However, if it persists and has a positive effect on fitness, natural selection will increase its frequency and may ultimately fix it in the population [40]. Monogenic traits are not very common and are normally linked to responses to biotic stresses (e.g., resistance to pests and pathogens). On the contrary, traits related to responses to abiotic stresses or with the start of seasonal physiological processes are largely polygenic [41]. In simple traits, each gene contributes with a small effect to the phenotype, whereas in complex traits, the phenotype depends on a network of genes that interact in a non-reciprocal way (i.e., the expression of one gene allows or inhibits the expression of others). How genetic adaptation of polygenic traits takes place is still under debate, but according to the most accepted model, populations adapt to the new conditions through small simultaneous changes in the allelic frequencies of many loci, until a new optimum phenotype is reached [40,42]. Furthermore, several studies have shown that the adaptation of polygenic traits not only takes place through changes in allelic frequencies but also through changes in epistatic effects [43,44].

Other factors to consider in the process of evolutionary adaptation are pleiotropy and gene linkage. Pleiotropy takes place when a single gene affects several seemingly unrelated phenotypic traits. From a theoretical viewpoint, the pleiotropic architecture can facilitate rapid adaptation if selection shifts all affected traits to their optimum. On the contrary, it can hamper evolution if it has a positive effect on some traits and a negative effect on others [45]. Similarly, gene linkage can also promote or hamper adaptation [46].

Finally, to this complex network of gene interactions, we must add the epigenetic changes that take place as a response to various environmental factors and that, in some cases, can be transmitted through several generations. Thus, phenotypic plasticity also contributes to adaptation [47,48,49].

### 2.2. Evolutionary Rescue

When environmental conditions change and phenotypic plasticity is not enough to maintain absolute fitness values, populations experience a declining trend that may lead to extinction. However, theory predicts that populations may recover if allele combinations that improve individual fitness to the new environmental conditions are available, or if advantageous mutations arise, in a process known as evolutionary rescue [50].

The probability of an evolutionary rescue depends on numerous factors, including the severity and speed of environmental change and the initial characteristics of the population (mainly population size and standing genetic diversity) [36,51]. If the rate of environmental change is too fast, the time available for natural selection to take place and increase the frequency of favorable genotypes may not be enough to facilitate population recovery. Similarly, evolutionary rescue is improbable in small populations, which is common for many threatened species. On one hand, small populations are associated with less genetic diversity and, thus, lower probability of occurrence of favorable alleles. On the other hand, if favorable alleles are found, the risk of losing them through genetic drift is greater, even if they are of adaptive value [38,52]. Interestingly, a number of theoretical models and experimental studies have highlighted the impact of migration on the probability of evolutionary rescue [37,53,54,55]. For example, Tomasini and Peischl [56] have showed in a two-deme model that migration favors evolutionary rescue when (a) environmental change occurs slowly across two populations (which leaves time for the second population to serve as a migration source), (b) the new environment is very harsh and (c) rescue mutations are strongly beneficial in the new environment. These conditions ensure that the rescue mutations can spread easily in the new environment.

### 2.3. Facilitated Adaptation

**Figure 1:**
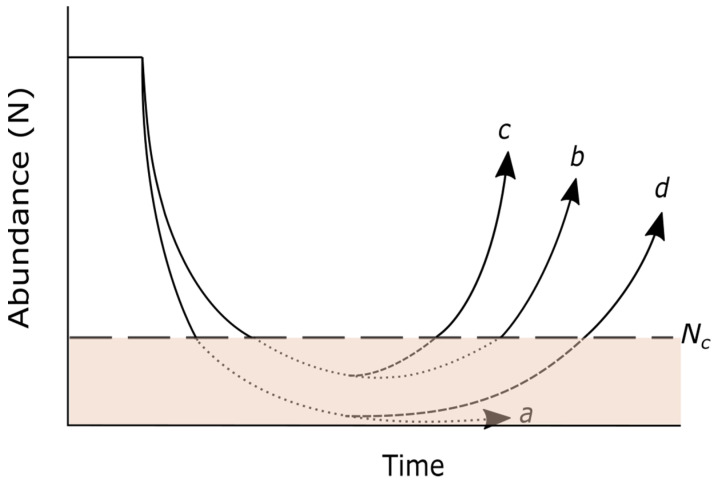
Conceptual diagram of facilitated adaptation. Population abundance (N) and extinction risk in the presence or absence of evolution. Following the conventions of Gomulkiewicz and Holt [57], growth is density independent, and N_C_ represents a threshold abundance below which the possibilities of extinction are high. Dashed portions of curves represent time at greatest risk of extinction for populations faced with a shift in their selective environment: (a) population declines to extinction in the absence of relevant standing genetic variation or new beneficial mutations; (b) evolution is insufficient to prevent the population from entering the extinction risk zone, but allows the population to grow out of that zone if new beneficial allelic combinations or mutations arise; (c) reinforcement with pre-adapted individuals provides beneficial alleles; this allows the population to avoid extinction and to reduce the recovery time; (d) reinforced individuals provide beneficial alleles, but also maladapted alleles.

Facilitated adaptation, according to Thomas et al. [32], is a strategy to promote the evolutionary rescue of populations affected by climate change by introducing individuals with adaptive alleles. As mentioned before, natural selection can only operate on standing genetic variation. Thus, the objective is to add beneficial alleles to accelerate or make possible the adaptation process (Figure 1).

Under this definition, facilitated adaptation can be approached in two different ways. If adaptive alleles are present in the focal population, it involves reinforcement of the population with more pre-adapted individuals. When adaptive alleles are not present in the population, it is necessary to introduce those alleles through gene flow. The source of alleles with adaptive value is an important issue to consider because it conditions the method to be used to include them in the focal population. The second approach implies to generate variation de novo, that is, new alleles or allelic combinations. For this purpose, many mechanisms exist such as selective breeding, genome editing techniques, and hybridization. Selective breeding has been used for centuries in agriculture to obtain improved cultivars. It involves the selection of individuals that breed, allowing to breed only those that have the desired traits (positive selection) or avoiding the reproduction of those with undesired traits (negative selection). Thus, variation arises naturally by shuffling of parental genes. Genome editing techniques make targeted changes to the genome of an organism, predominantly by using site-specific endonucleases such as CRISPR-Cas9. In this case, novel variation is due to mutations in specific genes (punctual mutations, insertions or deletions) or chromosomal rearrangements [58]. Hybridization between species could also provide new allelic combinations [59]. However, it entails the loss of identity of the target species, and therefore, its use departs from the principles of facilitated adaptation. A radically different way of obtaining individuals with the desired phenotype would imply altering gene expression instead of gene composition. Recent studies have shown that some epigenetic marks are mitotically stable and can even be heritable along several generations [60]. Epigenetics might then be used as a tool to obtain pre-adapted individuals, not only in clonally reproducing perennial species, but also in sexually reproducing species [61].

From a practical point of view, facilitated adaptation is a type of genetic translocation focused on increasing adaptive potential to respond the climate change. Therefore, it should not be confused with a genetic rescue whose aim is to overcome the reduction in fitness derived from inbreeding depression. On the other hand, terms such as “adaptive introgression”, “targeted gene flow” or “assisted gene flow” would be considered under the umbrella of facilitated adaptation as they represent different ways to introduce beneficial alleles to encourage the evolutionary adaptation to environmental changes, whereas “assisted evolution”, as defined by van Oppen et al. [62], would encompass facilitated adaptation (Table 1).

## 3. Climate-Change Adaptive Traits

A key aspect for implementing an adaptive facilitation strategy is the identification of the phenotypic traits that may confer a focal population the required adaptation to climate change. The challenges that can be brought up by climate change are diverse and span along a wide range of direct and indirect abiotic and biotic limiting factors that will vary among species (reviewed by Becklin et al. [66] and Hamann et al. [67]). Therefore, the phenotypic traits that are likely to grant adaptation to climate change will also be numerous and, in many cases, species specific. Nevertheless, certain traits may play a more important and general role improving adaptation to climate change for a wide range of species.

A growing number of studies show that species are changing the phenology of their life cycles in response to climate change. Germination, leaf emergence, flowering and fruiting of many species from cold and temperate zones have advanced in concert with warming trends [68,69,70,71]. In some cases, it has been shown that these changes have a genetic base. For instance, Qian et al. [72] detected SNPs/indels located in three genes associated with flowering time regulation (*FLOWERING TIME LOCUS T*, *CONSTANS-LIKE PROTEIN 1*, and *VERNALIZATION-H2*) in populations of *Hordeum spontaneum* K. Koch from Israel sampled 28 years apart. In this case, natural selection would have favored early flowering because it allows the plants to complete their life-cycle before the onset of the dry period. Similarly, Franks et al. [11] also showed rapid changes in genes related to flowering time in two populations of *Brassica rapa* L. in response to a series of dry years, in which drought was particularly severe late in the growing season. Early-flowering genotypes can therefore be advantageous for annual plants in environments with short growing seasons that are ended by terminal drought (see Kooyers [73] for other strategies of drought response and associated phenotypes). The direction of selection for plants that are particularly sensitive to temperature cues, such as those at high latitudes or altitudes, is more difficult to predict because opposite drivers could be operating. Temperature increases in spring may lead to advancing flowering phenology, but warming in winter may delay the fulfillment of chilling requirements and thus lead to later onset of flowering [74].

Plants can also adapt to climate change by developing mechanisms to avoid and/or tolerate heat stress. For instance, in a common garden experiment on *Arabidopsis thaliana* (L.) Heynh. sampled across a latitudinal gradient, Hopkins et al. [75] found that genotypes from low latitudes had more erect leaves that genotypes from high latitudes, which is a way to reduce the leaf area that is exposed to heat from sunlight and, therefore, an adaptation to avoid heat stress (see Wahid et al. [76] for other morphological and physiological adaptations). In another experimental study, Tonsor et al. [77] found variation of ATHsp101 expression in *A. thaliana* plants from different latitudes, and revealed that AtHsp101 content had an effect on fitness. AtHsp101 is an essential protein to confer heat tolerance in *A. thaliana* [78,79], whose expression levels correspond to different allelic forms [80]. Thus, natural populations would have evolved different patterns of AtHsp101 expression in response to different patterns of heat stress. Similarly, Barua et al. [81] related the content of Hsps (other heat-shock proteins) and thermotolerance in five populations of *Chenopodium album* L. from contrasting thermal environments. Interestingly, they showed that plants native to more stressful habitats (i.e., with higher mean temperature and more frequent exposure to extreme temperatures) had lower levels of Hsp accumulation and induced thermotolerance, suggesting a greater reliance on basal mechanisms of heat stress response. These results indicate that genotypes of a species from warmer more-thermally variable habitats may be better adapted to climate change than those from cooler, more-thermally stable habitats, but also that the ability to respond may be limited in populations where temperatures are close to the limit of thermal tolerance, as found in populations occurring at low latitudes [82,83].

With this variety of responses one can ask whether the type of adaptive response can be somewhat predictable at the species level or depends on each population. In this context, a genomic study on local adaptation of *Pinus contorta* Douglas and *Picea glauca* (Moench) Voss showed that great part of the genetic variation attributable to changing climatic conditions corresponded to the same genes, suggesting that the adaptive response would be constrained to certain key genes [84]. However, Sacristán et al. [85] observed, in an experiment of artificial selection with *Lupinus angustifolius* L., that selection for early flowering was associated with changes in different functional traits (plant height, biomass, shoot growth, specific leaflet area and leaflet dry matter content) and that the effects of these changes depended on the population of origin.

## 4. A Methodological Proposal for Facilitated Adaptation

**Figure 2:**
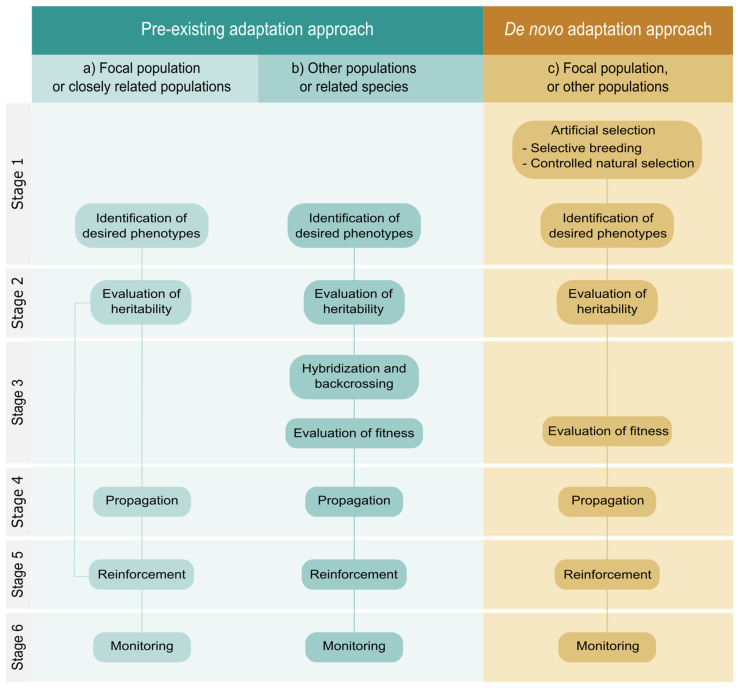
Framework for the implementation of the facilitated adaptation strategy, with alternative procedures depending on the pre-existing genetic variation. (**a**) Pre-adapted individuals from the same population or from other genetically close populations are multiplied and translocated to the focal population. (**b**) Pre-adapted individuals from genetically distinct populations or from related species are crossed with individuals from the focal population. Resulting offspring is repeatedly backcrossed with individuals from the focal population to eliminate undesirable traits while retaining the targeted trait. Next, the progeny is multiplied and translocated to the focal population. (**c**) Individuals from the focal population or from other populations are selected and crossed repeatedly to obtain individuals with the desired traits. New allelic combinations could also be promoted by submitting individuals to stress conditions for several generations. Once fitness has been evaluated, pre-adapted individuals are multiplied and translocated. All procedures require a final stage of demographic monitoring.

Herein, we propose two approaches that can be followed to implement the facilitated adaptation strategy. The first one (hereafter, *pre-existing adaptation approach*) is based on using pre-adapted genotypes existing in the focal population, in other populations, or even in closely related species. The second approach (hereafter, *de novo adaptation approach*) aims to generate new pre-adapted genotypes from the diversity present in the species by promoting crosses that favor genetic recombination (Figure 2). Although both approaches share some stages and techniques, they are conceptually different. This is an important issue because it implies that the difficulties and associated risks are also different. In both approaches, it is necessary to previously identify the cause of population decline, as well as the developmental stage on which facilitated adaptation will have the greatest effect. To obtain this basic information, manipulation experiments [86], demographic models [87,88] and structural equation models [89] can be used. Once the pre-adapted genotypes have been identified and propagated, the reinforcement of these plants should take place preferably before the target population size goes below the minimum viable population size and enters a status of high risk of extinction (Figure 1).

### 4.1. Pre-Existing Adaptation Approach

**Stage 1: Identification of the desired phenotype**. Taking into account the inherent stochasticity of climatic conditions through time in nature and the existence of microclimates [90], it is possible that the focal population may already contain individuals that have a phenotype that will allow them to adapt to future environmental conditions (for example, early-flowering or thermotolerant individuals). Therefore, the first step in this stage is to assess the variability of the trait of interest in the focal population and to check if there are individuals with the desired phenotype. If such individuals cannot be found in the focal population, they can be searched in other populations. In that case, GIS-based environmental maps can be useful to identify candidate populations, even though the correlation between phenotypic traits and environmental variables does not always imply a causal selection-based effect [91]. Predictive characterization can also be helpful to identify sets of genotypes with a high probability of containing ‘target’ traits [92]. A third approach to identify pre-adapted individuals is using landscape genomics, an emerging research field that aims to identify changes in the genome directly resulting from local environmental factors [93,94,95,96].

**Stage 2: Check the heritability of the desired traits**. Once the individuals with the desired traits have been identified, it is necessary to assess the narrow-sense heritability of such traits (i.e., the ratio of additive variance over phenotypic variance). Different approaches can be taken to measure the narrow-sense heritability depending on the type of data that can be obtained, whether the data are gathered in experimental settings using common garden experiments or in natural settings, and the generation time of the species (see Falconer and Mackay [97] for details). When the generation time of the species is high, it may be more efficient timewise to use estimations based on sibling analysis, where the phenotypic resemblance among relatives versus non-relatives is measured in a single generation. Otherwise, different approaches involving phenotypic measurements of parents and offspring or different individuals of known relatedness over two or more generations can be used [98]. Depending on the type of data gathered, parent–offspring regression, full-sib/half-sib analysis and the animal model can be applied. The latter provides the most accurate estimations of heritability as it consists of a mixed model that can account for various other sources of resemblance such as maternal and common environment effects [99]. If heritability were low, the feasibility of this approach is compromised. In such case, the de novo adaptation approach could be considered as an alternative to increase heritability.

**Stage 3: Gene introgression**. If pre-adapted individuals are identified in the focal population or in a near-by population of the same lineage, they can be propagated directly (Figure 2a). However, if the pre-adapted individuals are identified in genetically distinct populations or even in other closely related species, the adaptive allele gene combinations will have to be introduced through crossings with individuals of the focal population (Figure 2b). To recover the genetic background of the focal population, the offspring will be successively backcrossed with individuals of the focal population while selecting those individuals that retain the desired traits. In practice, after the fourth backcrosses, the progeny will closely resemble the focal population and express the desired donor trait [97]. Fitness consequences of introgression should be tested for at least two generations to control the risk of outbreeding depression on the focal population. For the same reason, individuals from populations with fixed chromosomal differences should be avoided (see decision tree to predict the risk of outbreeding depression devised by Frankham et al. [100]).

**Stage 4: Propagation of pre-adapted individuals**. The implementation of this stage and its limitations will be conditioned by the breeding system of each species. Clonal reproduction and self-pollination facilitate the maintenance through generations of the desired phenotypes. However, propagation through cross-pollination will require the selection of pre-adapted individuals in each generation. In any case, it is important that the procedure not only focuses on obtaining a set number of individuals with the desired traits, but also that all pre-adapted individuals identified at the start of the process proportionally contribute to the progeny to be used in the reinforcement.

**Stage 5: Reinforcement of the focal population**. Pre-adapted individuals propagated ex situ are translocated to the focal population. This way the number of individuals with greater fitness under the new environmental conditions is increased along with the frequency of adaptive alleles in the population. If individuals are obtained via gene introgression, a pre-release assessment is advisable to test their fitness and the effect of genotype-by environment interaction in natural conditions. As in any other reinforcement operation, relevant decisions will have to be made concerning the number and age of the individuals to establish, spatial location, etc. (see [101,102]). In addition to these recommendations, trait-specific properties (selection differential and heritability) must be taken into account to decide the proportion of introduced individuals. Elasticity analyses [103,104] can help to predict the impact of trait variation on growth rate and, therefore, the intensity of selection on the trait needed to obtain the desired results. Population models such as those developed by Kelly and Phillips [105] may also be a useful tool to determine the number of individuals to reinforce and the best time to do it.

**Stage 6: Demographic and genetic monitoring**. In this last stage, a demographic monitoring focusing on per capita growth rate will be carried out to assess the intervention success. The population will be monitored until the vital rates are stabilized and the population recovers a population size large enough to sustain the effects of demographic and environmental stochasticity. This may entail many years, and it will greatly depend on the severity of climate change and the generation time of the species, but it should last a minimum of 10 years. In annual and biennial plants, monitoring should take place every year or every other year. In perennial plants, with a higher generation time, the periodicity of the monitoring could be reduced to one survey every 5 years. The evaluation method will implement a population viability analysis based on count data or structured population data that will enable us to infer the demographic trends [106]. Genetic monitoring to assess the degree of allele introgression in the focal population and other genetic variation effects could also provide relevant information and would be especially important if demographic monitoring does not detect an improvement in the demographic trend [107]. Population size, number of introduced individuals, species breeding system and growth rate will be some of the factors that will condition the speed and intensity of introgression. In parallel, the impact of the operation on the host ecosystem should also be assessed, especially if the target taxon is a dominant or keystone species.

### 4.2. De Novo Adaptation Approach

**Stage 1: Artificial selection**. One way to generate the target phenotype is through selective breeding by favoring crossings between selected individuals for several generations to create new allelic combinations in different genes. The resulting new genotypes can then be subject to mass selection, i.e., only those individuals above or below a given trait value are chosen as parents for the next generation [108]. The threshold trait value can be a fixed value over successive generations (selection by constant truncation) or a fixed percentage of the population representing the highest or lowest value of the selected trait (selection by proportional truncation). In selection by constant truncation, the intensity of selection decreases with time, as a greater frequency of the population exceeds the fixed truncation point. In selection by proportional truncation, instead, the intensity of selection is constant [109]. Another aspect to consider is that selection by constant truncation can greatly reduce genetic diversity if the number of individuals selected in the first generation is low. Regardless of the method, individuals used for the crossings may originate from the focal population or from other populations.

Another way to generate and select new allelic combinations that deliver the target phenotype involves allowing free mating among a sample of individuals from the focal population or from other populations and subjecting them to increasing stress conditions related to the direction of phenotypic change for several generations. In this case, selection is not based in a particular value of a desired trait but on the ability of the experimental populations to survive and reproduce under the stress conditions provided. This method, named “controlled natural selection”, is being used to obtain coral individuals adapted to thrive in warm waters [110].

Depending on the breeding system and life cycle of the species, the genetic basis (monogenic vs. polygenic) of the targeted trait and the procedures available to characterize the trait, a range of more specific selection methodologies developed by plant breeders can be considered for implementation [111,112].

**Stage 2: Check the heritability of the desired trait**. When the offspring have been obtained under stressful conditions, additional experiments are required to ensure they have adapted rather than acclimated. Thus, trait heritability must be estimated in a way similarly to that indicated in the pre-existing adaptation approach.

**Stage 3: Assessment of effects in other traits**. Artificial selection on the trait of interest may affect other traits due to pleiotropy and gene linkage [113]. For example, Galloway and Burgess [114] selected for three years early and late flowering individuals of *Campanulastrum americanum* (L.) Small and observed that other phenological traits related to reproduction were also modified. Specifically, seed dispersal date was more advanced in early flowering plants and became more retarded in late flowering plants, subsequently affecting the annual or biennial behavior of progeny.

The purpose of this stage is to identify potential changes in other traits that might have a negative effect on the fitness of the individual or its progeny. The presence of a strong correlation with another trait that changes in the direction of reducing the fitness of the individual may completely invalidate this approach. In these cases, the availability of genetic markers that can detect at early life stages the target phenotype of the individuals will improve the efficacy of this stage. On the other hand, the possibility of using annotated genomes of phylogenetically close species and the advance in the understanding of gene regulatory networks in model species will help predict the traits that can be affected by artificial selection and help us to assess the consequences (e.g., [115]).

**Stages 4, 5 and 6** would be carried out in a similar way to what has been presented for the pre-existing adaptation approach. Before translocation, the evaluation of crossings with individuals from the local population would be advisable to detect a possible loss of fitness by outbreeding depression [116].

## 5. Risks and Difficulties of Facilitated Adaptation

Facilitated adaptation, as any other conservation strategy, is not risk exempt. Some precautionary principles to minimize the risk of reducing genetic diversity and inducing unwanted effects on non-targeted traits have already been mentioned in the description of the methodology. Here, we discuss other aspects to take into account.

*Loss of genetic integrity*. It is evident that the introduction of artificially selected individuals comports a change in the genetic composition of the focal population. Many conservationists assume this loss of genetic integrity as a trade-off to increase the evolutionary resilience of the species and to reduce its extinction risk [31,63]. Other scientists do not even ponder this as a risk, because gene flow is one of the main evolutionary forces [117] and new hybridization zones are being generated as a result of climate change (e.g., [118]).

*Loss of local adaptations*. The reinforcement with individuals from other populations can dilute local adaptations non-related to climatic factors that could compromise the short- or long-term viability of the population. One example of this type of adaptations is the polymorphism in the genes involved in the circadian regulation of several biological processes that are controlled by photoperiod, such as flowering onset [119]. Since photoperiod varies with latitude, species with wide latitudinal ranges may be more vulnerable to this risk. Thus, it is advisable that individuals used in crosses originate from similar latitudes. Other examples of local adaptations non-related to climatic factors are found in adaptations originated from biotic interactions, such as defense mechanisms against herbivores. For instance, in *Arabidopsis thaliana*, polymorphism of *GS-Elong* locus, involved in glucosinolate synthesis, has been related to the abundance of two aphid species along a longitudinal cline across Europe [120]. Nevertheless, several studies in adaptation genomics carried out in recent years indicate that this risk may be overvalued, because most adaptive traits are the result of the interaction of many genes, each one with small effects [121].

*Loss of natural conditions*. There is also the possibility of losing adaptation to natural conditions as a result of the maintenance of selected individuals at ex situ conditions over several generations [122,123]. As the negative effects dramatically increase with the number of generations grown at ex situ conditions, this risk can be minimized by using individuals of the first generations [124]. Anyway, the debate on whether it is advisable to take the risk of losing the naturalness of ecosystems and the genetic integrity of species and populations to fight the threat of extinction is still open [125].

*Pathogens or pests*. Another risk, shared with assisted migration, is the possibility of involuntarily introducing pathogens or pests in the target population. This risk can be minimized by the implementation of exhaustive controls and subjecting individuals to a quarantine period before translocation to the focal population [101]. Individuals that are susceptible to diseases present in the focal population should not be introduced.

*Ecological risks*. Although facilitated adaptation does not imply the introduction of a new species in the ecosystem, the genetic variation resulting from the introduction of selected individuals in the focal population could change the composition of associated communities, and even modify ecosystem processes. Crutsinger et al. [126], for example, showed experimentally that increasing population genotypic diversity in a dominant old-field plant species, *Solidago altissima* L., determined arthropod richness and community structure, and increased net primary productivity. Other examples with *Oenothera biennis* L. [127] or *Ammophila breviligulata* Fern. [128] also demonstrate that genetic variation in a population can have community-level consequences. Therefore, in organisms with high interspecific indirect genetic effects, the use of facilitated adaptation can be considered a risk if it implies loss of associated biodiversity or significant changes on species interactions.

In this context, it is worth considering the possibility that translocated individuals could acquire an invasive behavior at the receptor site as a result of their improved adaptive capabilities. According to the current hypotheses on the main factors that promote the invasive behavior in a species [129], this scenario is very unlikely to happen because translocated individuals would occupy the same ecological niche than the native individuals of the focal population.

*Technical difficulties*. The reproductive characteristics and life history of certain target species can bring technical difficulties. For instance, crosses by hand are difficult to make in species with very small flowers or with long generation times. In such cases, the procedures that require several backcrosses to eliminate undesirable traits or directed crosses would not be advisable.

Phenological mismatches could also represent a difficulty for the procedures that require crosses between individuals [130]. Differences in flowering time, for example, would imply to pollinate by hand with preserved pollen grains.

Small populations could require greater effort to introduce beneficial alleles. Thus, repeated introductions could be necessary to counteract the loss of these alleles by genetic drift.

## 6. Concluding Remarks

Facilitated adaptation is a recent conservation strategy that emerges as a response to the increasing threats that climate change brings on many species. It could be adopted as a complementary measure to other type of actions oriented to reduce or delay changes in the ecosystem. It is important to note that this strategy cannot be widely applied, as it is costly, and its efficacy depends on the species (Box 1) and the particular conditions residing in the focal population [85,131]. In addition, for most plant species we still lack a comprehensive knowledge of the genes and metabolic networks associated with the generation of adaptive phenotypic traits, as well as the existing constraints and inter-dependencies associated between traits. Nevertheless, technical advances of the last decade are enabling a better understanding of the genetic basis of adaptive traits and the subjacent mechanisms that operate at short and long terms. This opens the possibility to raise new procedures to have a better control over the associated risks. In this sense, the following are is especially relevant: (i) the recent theory of polygenic adaptation, which is advancing toward models that integrate epistatic processes as a second source of variation; (ii) the findings in the field of epigenetics that have shown that adaptation is not solely dependent upon variation at DNA-level [132,133]; and (iii) the CRISPR/Cas system of genome editing, which allows us to study gene function in genes of non-model species, as well as the role that genetic variants play in the adaptation to environment (e.g., [134]). On the other hand, as genomic technologies are becoming less costly, DNA-based selection could be used to improve the efficiency of both approaches [135]. In particular, genomic selection decreases the time required to complete a breeding cycle by selecting the progeny in the early stages or before being tested in field experiments, which is especially relevant for long-lived trees. Furthermore, it allows scientists to overcome the difficulties related to the evaluation of complex and low heritable traits [136].

Box 1 **Ideal features of plant species that would be good candidates for facilitated adaptation.**

  *High-risk species*. Candidate species would correspond to species highly vulnerable to climate change, such as those occurring in tundra, alpine and Mediterranean environments.

  *Degree of adaptive plasticity.* Those species with a high degree of adaptive plasticity may not require facilitated adaptation. The candidates would be species that do not have an inherent high degree of adaptive plasticity.

  *Dispersal efficiency*. Those species with low dispersal efficiency cannot effectively migrate and would be candidates for facilitated adaptation.

  *Demographic characteristics*. Candidate populations would be those with low effective population size and low connectivity with other populations, who have few chances of experiencing a successful natural process of evolutionary rescue.

  *Degree of heterogeneity of the environment in which a species occurs*. Candidate species would ideally have a distribution range that covers a highly heterogeneous environment, and thus, they would be more likely to contain local adaptation processes that may provide the proper source that holds the genotype adapted to climate change.

  *Length of generation time*. Candidate species would have a short generation time, thus facilitating the development of selection cycles in a reasonably short period. Nevertheless, long-lived species can also benefit of facilitated adaptation if genomic selection is used instead of conventional phenotypic selection.

  *Reproductive rates*. Candidate species would ideally have high reproductive rates, which would facilitate the selection process and the obtention of a great number of individuals with the targeted genotype.

  *Plant traits*. Target traits should be those that are most critical for climate change adaptation regardless of whether they are fixed traits or labile traits. Qualitative traits, which are determined by one or few genes, would be more amenable for adaptive facilitation than quantitative traits. Once again, it would be the traits at the stage of development that is more critically limited by the environmental conditions altered by climate change.

The different procedures that have been described in this article to implement facilitated adaptation vary in the degree of complexity and associated risks. The procedures based on the reinforcement of the focal population with pre-adapted individuals originated from the same population or genetically close populations are simpler and have fewer associated risks than those that require crossings with individuals of genetically diverging populations or closely related species. Furthermore, the procedures based in the use of pre-adapted individuals are simpler than those that involve the generation of new allelic combinations in different genes. Theoretically, the implementation of facilitated adaptation will be easier in species with large population sizes [15,137] or that occur in areas with high environmental variation (either spatial, temporal or both) [138].

The uncertainty originated from potential genetic problems associated with other traits should be maximally reduced through previous experimentation with the focal species, involving studies on the epistatic and pleiotropic interactions of the target genes and assessments of the existing correlations among the target and most important functional traits.

## Figures and Tables

**Table 1:** Terms and definitions found in the literature to describe the introduction of beneficial alleles in a population to promote the evolutionary adaptation to climate change.

Term	Definition	Reference
*Adaptive introgression*	Movement of genetic material from the genome of one species into the genome of another through repeated interbreeding for increasing species’ ability to respond to a changing climate.	[63]
*Assisted evolution*	Any approach that accelerates the rate of naturally occurring evolutionary processes.	[62]
*Assisted gene flow*	The managed movement of individuals or gametes between populations within species ranges to mitigate local maladaptation in the short and long term.	[31]
*Facilitated adaptation*	Intervention to rescue a target population or species by endowing it with adaptive alleles, or gene variants, using genetic engineering.	[32]
*Genetic adaptation*	Genetic translocation to introduce new alleles for traits important for environmental change.	[64] ^1^
*Targeted gene flow*	Individuals that are pre-adapted to future conditions are translocated to increase the adaptive capacity of another population.	[65] ^1^

^1^ The authors also include the introduction or movement of particular variants to areas outside the current range, which would be an assisted migration under the taxonomy of terms followed in this review.

## Data Availability

No new data were created or analyzed in this study. Data sharing is not applicable to this article.

## References

[1] Scheffers B.R., De Meester L., Bridge T.C.L., Hoffmann A.A., Pandolfi J.M., Corlett R.T., Butchart S.H.M., Pearce-Kelly P., Kovacs K.M., Dudgeon D. (2016). The broad footprint of climate change from genes to biomes to people. Science.

[2] Wiens J.J. (2016). Climate-related local extinctions are already widespread among plant and animal species. PLoS Biol..

[3] Bertin R.I. (2008). Plant phenology and distribution in relation to recent climate change. J. Torrey Bot. Soc..

[4] Chen I.C., Hill J.K., Ohlemüller R., Roy D.B., Thomas C.D. (2011). Rapid range shifts of species associated with high levels of climate warming. Science.

[5] Lenoir J., Svenning J.C., Levin S.A. (2013). Latitudinal and elevational range shifts under contemporary climate change. Encyclopedia of Biodiversity.

[6] Freeman B.G., Lee-Yaw J.A., Sunday J.M., Hargreaves A.L. (2018). Expanding, shifting and shrinking: The impact of global warming on species’ elevational distributions. Glob. Ecol. Biogeogr..

[7] Cheplick G.P. (2022). Philomatry in plants: Why do so many species have limited seed dispersal?. Am. J. Bot..

[8] Honnay O., Verheyen K., Butaye J., Jacquemyn H., Bossuyt B., Hermy M. (2002). Possible effects of habitat fragmentation and climate change on the range of forest plant species. Ecol. Lett..

[9] Christmas M.J., Breed M.F., Lowe A.J. (2016). Constraints to and conservation implications for climate change adaptation in plants. Conserv. Genet..

[10] Anderson J.T., Inouye D.W., McKinney A.M., Colautti R.I., Mitchell-Olds T. (2012). Phenotypic plasticity and adaptive evolution contribute to advancing flowering phenology in response to climate change. Proc. R. Soc. B Biol. Sci..

[11] Franks S.J., Kane N.C., O’Hara N.B., Tittes S., Rest J.S. (2016). Rapid genome-wide evolution in *Brassica rapa* populations following drought revealed by sequencing of ancestral and descendant gene pools. Mol. Ecol..

[12] Rauschkolb R., Henres L., Lou C., Godefroid S., Dixon L., Durka W., Bossdorf O., Ensslin A., Scheepens J.F. (2022). Historical comparisons show evolutionary changes in drought responses in European plant species after two decades of climate change. Basic Appl. Ecol..

[13] Wang Y., Chen G., Zeng F., Han Z., Qiu C.-W., Zeng M., Yang Z., Xu F., Wu D., Deng F. (2023). Molecular evidence for adaptive evolution of drought tolerance in wild cereals. New Phytol..

[14] Brunet J., Larson-Rabin Z. (2012). The response of flowering time to global warming in a high-altitude plant: The impact of genetics and the environment. Botany.

[15] Hoffmann A.A., Sgrò C.M., Kristensen T.N. (2017). Revisiting adaptive potential, population size, and conservation. Trends Ecol. Evol..

[16] Urban M.C. (2015). Accelerating extinction risk from climate change. Science.

[17] Jump A.S., Peñuelas J. (2005). Running to stand still: Adaptation and the response of plants to rapid climate change. Ecol. Lett..

[18] Skelly D.K., Joseph L.N., Possingham H.P., Freidenburg L.K., Farrugia T.J., Kinnison M.T., Hendry A.P. (2007). Evolutionary responses to climate change. Conserv. Biol..

[19] Hoffmann A.A., Sgrò C. (2011). Climate change and evolutionary adaptation. Nature.

[20] Keeley A.T.H., Ackerly D.D., Cameron D.R., Heller N.E., Huber P.R., Schloss C.A., Thorne J.H., Merenlender A.M. (2018). New concepts, models, and assessments of climate-wise connectivity. Environ. Res. Lett..

[21] Hällfors M.H., Vaara E.M., Hyvärinen M., Oksanen M., Schulman L.E., Siipi H., Lehvävirta S. (2014). Coming to terms with the concept of moving species threatened by climate change—A systematic review of the terminology and definitions. PLoS ONE.

[22] Hunter M.L. (2007). Climate change and moving species: Furthering the debate on assisted colonization. Conserv. Biol..

[23] McLachlan J.S., Hellmann J.J., Schwartz M.W. (2007). A framework for debate of assisted migration in an era of climate change. Conserv. Biol..

[24] Ricciardi A., Simberloff D. (2009). Assisted colonization is not a viable conservation strategy. Trends Ecol. Evol..

[25] Carrete M., Tella J.L. (2012). Is assisted colonization feasible? Lessons from past introductions. Front. Ecol. Environ..

[26] Dukes J.S., Mooney H.A. (2004). Disruption of ecosystem processes in western North America by invasive species. Rev. Chil. Hist. Nat..

[27] Scarela R., Genovesi P., Essl F., Rabitsch W. (2012). The Impacts of Invasive Alien Species in Europe.

[28] Williams M.C., Wardle G.M. (2007). *Pinus radiata* invasion in Australia: Identifying key knowledge gaps and research directions. Austral Ecol..

[29] Kew R. (2016). The State of the World’s Plants Report—2016.

[30] Sgrò C.M., Lowe A.J., Hoffmann A.A. (2011). Building evolutionary resilience for conserving biodiversity under climate change. Evol. Appl..

[31] Aitken S.N., Whitlock M.C. (2013). Assisted gene flow to facilitate local adaptation to climate change. Annu. Rev. Ecol. Evol. Syst..

[32] Thomas M.A., Roemer G.W., Donlan C.J., Dickson B.G., Matocq M., Malaney J. (2013). Gene tweaking for conservation. Nature.

[33] Ahuja I., de Vos R.C.H., Bones A.M., Hall R.D. (2010). Plant molecular stress responses face climate change. Trends Plant Sci..

[34] Franks S.J., Hoffmann A.A. (2012). Genetics of climate change adaptation. Annu. Rev. Genet..

[35] Vanwallendael A., Soltani A., Emery N.C., Peixoto M.M., Olsen J., Lowry D.B. (2019). A molecular view of plant local adaptation: Incorporating stress-response networks. Annu. Rev. Plant Biol..

[36] Carlson S.M., Cunningham C.J., Westley P.A.H. (2014). Evolutionary rescue in a changing world. Trends Ecol. Evol..

[37] Hufbauer R.A., Szűcs M., Kasyon E., Youngberg C., Koontz M.J., Richards C., Tuff T., Melbourne B.A. (2015). Three types of rescue can avert extinction in a changing environment. Proc. Natl. Acad. Sci. USA.

[38] Stewart G.S., Morris M.R., Genis A.B., Szűcs M., Melbourne B.A., Tavener S.J., Hufbauer R.A. (2017). The power of evolutionary rescue is constrained by genetic load. Evol. Appl..

[39] Olson-Manning C.F., Wagner M.R., Mitchell-Olds T. (2012). Adaptive evolution: Evaluating empirical support for theoretical predictions. Nat. Rev. Genet..

[40] Pritchard J.K., Pickrell J.K., Coop G. (2010). The genetics of human adaptation: Hard sweeps, soft sweeps, and polygenic adaptation. Curr. Biol..

[41] Panigrahi J., Mishra R.R., Sahu A.R., Rath S.C., Kole C.R., Rout G.R., Das A.B. (2013). Marker-assisted breeding for stress resistence in crop plants. Molecular Stress Physiology of Plants.

[42] Pritchard J.K., Di Rienzo A. (2010). Adaptation—not by sweeps alone. Nat. Publ. Gr..

[43] Csilléry K., Rodríguez-Verdugo A., Rellstab C., Guillaume F. (2018). Detecting the genomic signal of polygenic adaptation and the role of epistasis in evolution. Mol. Ecol..

[44] Fagny M., Austerlitz F. (2021). Polygenic adaptation: Integrating population genetics and gene regulatory networks. Trends Genet..

[45] Pavličev M., Cheverud J.M. (2015). Constraints evolve: Context dependency of gene effects allows evolution of pleiotropy. Annu. Rev. Ecol. Evol. Syst..

[46] Pepper J.W. (2003). The evolution of evolvability in genetic linkage patterns. BioSystems.

[47] Fox R.J., Donelson J.M., Schunter C., Ravasi T., Gaitán-Espitia J.D. (2019). Beyond buying time: The role of plasticity in phenotypic adaptation to rapid environmental change. Philos. Trans. R. Soc. B Biol. Sci..

[48] Thiebaut F., Hemerly A.S., Ferreira P.C.G. (2019). A role for epigenetic regulation in the adaptation and stress responses of non-model plants. Front. Plant Sci..

[49] Stajic D., Jansen L.E.T. (2021). Empirical evidence for epigenetic inheritance driving evolutionary adaptation. Philos. Trans. R. Soc. B Biol. Sci..

[50] Gomulkiewicz R., Shaw R.G. (2013). Evolutionary rescue beyond the models. Philos. Trans. R. Soc. Lond. B. Biol. Sci..

[51] Bell G. (2013). Evolutionary rescue and the limits of adaptation. Philos. Trans. R. Soc. B Biol. Sci..

[52] Bell G., Gonzalez A. (2009). Evolutionary rescue can prevent extinction following environmental change. Ecol. Lett..

[53] Bell G., Gonzalez A. (2011). Adaptation and evolutionary rescue in metapopulations experiencing environmental deterioration. Science.

[54] Schiffers K., Bourne E.C., Lavergne S., Thuiller W., Travis J.M.J. (2013). Limited evolutionary rescue of locally adapted populations facing climate change. Philos. Trans. R. Soc. Lond. B. Biol. Sci..

[55] Czuppon P., Blanquart F., Uecker H., Débarre F. (2021). The effect of habitat choice on evolutionary rescue in subdivided populations. Am. Nat..

[56] Tomasini M., Peischl S. (2020). When does gene flow facilitate evolutionary rescue?. Evolution.

[57] Gomulkiewicz R., Holt R.D. (1995). When does evolution by natural selection prevent extinction?. Evolution.

[58] Phelps M.P., Seeb L.W., Seeb J.E. (2020). Transforming ecology and conservation biology through genome editing. Conserv. Biol..

[59] Chan W.Y., Hoffmann A.A., van Oppen M.J.H. (2019). Hybridization as a conservation management tool. Conserv. Lett..

[60] Lind M.I., Spagopoulou F. (2018). Evolutionary consequences of epigenetic inheritance. Heredity.

[61] Rey O., Eizaguirre C., Angers B., Baltazar-Soares M., Sagonas K., Prunier J.G., Blanchet S. (2020). Linking epigenetics and biological conservation: Towards a conservation epigenetics perspective. Funct. Ecol..

[62] van Oppen M.J.H., Oliver J.K., Putnam H.M., Gates R.D. (2015). Building coral reef resilience through assisted evolution. Proc. Natl. Acad. Sci. USA.

[63] Hamilton J.A., Miller J.M. (2016). Adaptive introgression as a resource for management and genetic conservation in a changing climate. Conserv. Biol..

[64] Weeks A.R., Sgro C.M., Young A.G., Frankham R., Mitchell N.J., Miller K.A., Byrne M., Coates D.J., Eldridge M.D.B., Sunnucks P. (2011). Assessing the benefits and risks of translocations in changing environments: A genetic perspective. Evol. Appl..

[65] Kelly E., Phillips B.L. (2016). Targeted gene flow for conservation. Conserv. Biol..

[66] Becklin K.M., Anderson J.T., Gerhart L.M., Wadgymar S.M., Wessinger C.A., Ward J.K. (2016). Examining plant physiological responses to climate change through an evolutionary lens. Plant Physiol..

[67] Hamann E., Denney D., Day S., Lombardi E., Jameel M.I., MacTavish R., Anderson J.T. (2021). Plant eco-evolutionary responses to climate change: Emerging directions. Plant Sci..

[68] Fitter A.H., Fitter R.S.R. (2002). Rapid changes in flowering time in British plants. Science.

[69] Amano T., Smithers R.J., Sparks T.H., Sutherland W.J. (2010). A 250-year index of first flowering dates and its response to temperature changes. Proc. R. Soc. B Biol. Sci..

[70] Ellwood E.R., Temple S.A., Primack R.B., Bradley N.L., Davis C.C. (2013). Record-breaking early flowering in the eastern United States. PLoS ONE.

[71] Parmesan C., Hanley M.E. (2015). Plants and climate change: Complexities and surprises. Ann. Bot..

[72] Qian C., Yan X., Shi Y., Yin H., Chang Y., Chen J., Ingvarsson P.K., Nevo E., Ma X.F. (2020). Adaptive signals of flowering time pathways in wild barley from Israel over 28 generations. Heredity.

[73] Kooyers N.J. (2015). The evolution of drought escape and avoidance in natural herbaceous populations. Plant Sci..

[74] Hu X., Zhou W., Sun S. (2020). Responses of plant reproductive phenology to winter-biased warming in an alpine meadow. Front. Plant Sci..

[75] Hopkins R., Schmitt J., Stinchcombe J.R. (2008). A latitudinal cline and response to vernalization in leaf angle and morphology in *Arabidopsis thaliana* (Brassicaceae). New Phytol..

[76] Wahid A., Gelani S., Ashraf M., Foolad M.R. (2007). Heat tolerance in plants: An overview. Environ. Exp. Bot..

[77] Tonsor S.J., Scott C., Boumaza I., Liss T.R., Brodsky J.L., Vierling E. (2008). Heat shock protein 101 effects in *A. thaliana*: Genetic variation, fitness and pleiotropy in controlled temperature conditions. Mol. Ecol..

[78] Hong S.W., Vierling E. (2000). Mutants of *Arabidopsis thaliana* defective in the acquisition of tolerance to high temperature stress. Proc. Natl. Acad. Sci. USA.

[79] Queitsch C., Hong S.-W., Vierling E., Lindquist S. (2000). Heat shock protein 101 plays a crucial role in thermotolerance in *Arabidopsis*. Plant Cell.

[80] Kumar R., Khungar L., Shimphrui R., Tiwari L.D., Tripathi G., Sarkar N.K., Agarwal S.K., Agarwal M., Grover A. (2020). AtHsp101 research sets course of action for the genetic improvement of crops against heat stress. J. Plant Biochem. Biotechnol..

[81] Barua D., Heckathorn S.A., Coleman J.S. (2008). Variation in heat-shock proteins and photosynthetic thermotolerance among natural populations of *Chenopodium album* L. from contrasting thermal environments: Implications for plant responses to global warming. J. Integr. Plant Biol..

[82] Sastry A., Barua D. (2017). Leaf thermotolerance in tropical trees from a seasonally dry climate varies along the slow-fast resource acquisition spectrum. Sci. Rep..

[83] Sentinella A.T., Warton D.I., Sherwin W.B., Offord C.A., Moles A.T. (2020). Tropical plants do not have narrower temperature tolerances, but are more at risk from warming because they are close to their upper thermal limits. Glob. Ecol. Biogeogr..

[84] Yeaman S., Hodgins K.A., Lotterhos K.E., Suren H., Nadeau S., Degner J.C., Nurkowski K.A., Smets P., Wang T., Gray L.K. (2016). Convergent local adaptation to climate in distantly related conifers. Science.

[85] Sacristán-Bajo S., García-Fernández A., Lara-Romero C., Prieto-Benítez S., Tabarés P., Morente-López J., Rubio Teso M.L., Alameda-Martín A., Torres E., Iriondo J.M. (2023). Population origin determines the adaptive potential for the advancement of flowering onset in *Lupinus angustifolius* L. (Fabaceae). Evol. Appl..

[86] Reed P.B., Peterson M.L., Pfeifer-Meister L.E., Morris W.F., Doak D.F., Roy B.A., Johnson B.R., Bailes G.T., Nelson A.A., Bridgham S.D. (2021). Climate manipulations differentially affect plant population dynamics within versus beyond northern range limits. J. Ecol..

[87] Akçakaya H.R., Burgman M.A., Kindvall O., Wood C.C., Sjögren-Gulve P., Hatfield J.S., McCarthy M.A. (2004). Species Conservation and Management: Case Studies.

[88] Giménez-Benavides L., Albert M.J., Iriondo J.M., Escudero A. (2011). Demographic processes of upward range contraction in a long-lived Mediterranean high mountain plant. Ecography.

[89] Iriondo J.M., Albert M.J., Escudero A. (2003). Structural equation modelling: An alternative for assessing causal relationships in threatened plant populations. Biol. Conserv..

[90] Bramer I., Anderson B.J., Bennie J., Bladon A.J., De Frenne P., Hemming D., Hill R.A., Kearney M.R., Körner C., Korstjens A.H., Bohan D.A., Dumbrell A.J., Woodward G., Jackson M. (2018). Advances in monitoring and modelling climate at ecologically relevant scales. Advances in Ecological Research.

[91] Kozak K.H., Graham C.H., Wiens J.J. (2008). Integrating GIS-based environmental data into evolutionary biology. Trends Ecol. Evol..

[92] Thormann I., Parra-Quijano M., Endresen D.T.F., Rubio-Teso M.L., Iriondo M.J., Maxted N. (2014). Predictive Characterization of Crop Wild Relatives and Landraces.

[93] Storfer A., Patton A., Fraik A.K. (2018). Navigating the interface between landscape genetics and landscape genomics. Front. Genet..

[94] Browne L., Wright J.W., Fitz-Gibbon S., Gugger P.F., Sork V.L. (2019). Adaptational lag to temperature in valley oak (*Quercus lobata*) can be mitigated by genome-informed assisted gene flow. Proc. Natl. Acad. Sci. USA.

[95] Exposito-Alonso M., Gómez Rodríguez R., Barragán C., Capovilla G., Chae E., Devos J., Dogan E.S., Friedemann C., Gross C., Lang P. (2019). Natural selection on the *Arabidopsis thaliana* genome in present and future climates. Nature.

[96] Forester B.R., Landguth E.L., Hand B.K., Balkenhol N., Hohenlohe P.A., Rajora O.P. (2021). Landscape genomics for wildlife research. Population Genomics: Wildlife.

[97] Falconer D.S., Mackay T.F.C. (1996). Introduction to Quantitative Genetics.

[98] Postma E., Charmantier A., Garant D., Kruuk L.E.B. (2014). Four decades of estimating heritabilities in wild vertebrate populations: Improved methods, more data, better estimates?. Quantitative Genetics in the Wild.

[99] Kruuk L.E.B. (2004). Estimating genetic parameters in natural populations using the “animal model”. Philos. Trans. R. Soc. B Biol. Sci..

[100] Frankham R., Ballou J.D., Eldridge M.D.B., Lacy R.C., Ralls K., Dudash M.R., Fenster C.B. (2011). Predicting the probability of outbreeding depression. Conserv. Biol..

[101] IUCN/SSC (2013). Guidelines for Reintroductions and Other Conservation Translocations. Version 1.0.

[102] Maschinski J., Haskins K.E. (2012). Plant Reintroduction in a Changing Climate. Promises and Peril.

[103] van Tienderen P.H. (2000). Elasticities and the link between demographic and evolutionary dynamics. Ecology.

[104] Walsh B., Lynch M. (2018). Evolution and Selection of Quantitative Traits.

[105] Kelly E., Phillips B. (2019). How many and when? Optimising targeted gene flow for a step change in the environment. Ecol. Lett..

[106] Morris W.F., Doak D.F. (2002). Quantitative Conservation Biology: Theory and Practice of Population Viability Analysis.

[107] van Rossum F., Hardy O.J. (2022). Guidelines for genetic monitoring of translocated plant populations. Conserv. Biol..

[108] Acquaah G., Al-Khayri J.M., Jain S.M., Johnson D.V. (2015). Conventional plant breeding principles and techniques. Advances in Plant Breeding Strategies: Breeding, Biotechnology and Molecular Tools.

[109] Griffiths A.J.F., Miller J.H., Suzuki D.T., Lewontin R.C., Gelbart W.M. (2000). An Introduction to Genetic Analysis.

[110] Chakravarti L.J., Beltran V.H., van Oppen M.J.H. (2017). Rapid thermal adaptation in photosymbionts of reef-building corals. Glob. Chang. Biol..

[111] Acquaah G. (2012). Principles of Plant Genetics and Breeding.

[112] Orton T.J. (2020). Horticultural Plant Breeding.

[113] Chen Y., Lübberstedt T. (2010). Molecular basis of trait correlations. Trends Plant Sci..

[114] Galloway L.F., Burgess K.S. (2012). Artificial selection on flowering time: Influence on reproductive phenology across natural light environments. J. Ecol..

[115] Satake A., Kawagoe T., Saburi Y., Chiba Y., Sakurai G., Kudoh H. (2013). Forecasting flowering phenology under climate warming by modelling the regulatory dynamics of flowering-time genes. Nat. Commun..

[116] Frankham R., Ballou J.D., Ralls K., Eldridge M.D.B., Dudash M.R., Fenster C.B., Lacy R.C., Sunnucks P. (2017). Genetic Managment of Fragmented Animal and Plant Populations.

[117] Ellstrand N.C., Rieseberg L.H. (2016). When gene flow really matters: Gene flow in applied evolutionary biology. Evol. Appl..

[118] Gómez J.M., González-Megías A., Lorite J., Abdelaziz M., Perfectti F. (2015). The silent extinction: Climate change and the potential hybridization-mediated extinction of endemic high-mountain plants. Biodivers. Conserv..

[119] Inoue K., Araki T., Endo M. (2018). Circadian clock during plant development. J. Plant Res..

[120] Züst T., Heichinger C., Grossniklaus U., Harrington R., Kliebenstein D.J., Turnbull L.A. (2012). Natural enemies drive geographic variation in plant defenses. Science.

[121] Tigano A., Friesen V.L. (2016). Genomics of local adaptation with gene flow. Mol. Ecol..

[122] Ensslin A., Tschöpe O., Burkart M., Joshi J. (2015). Fitness decline and adaptation to novel environments in ex situ plant collections: Current knowledge and future perspectives. Biol. Conserv..

[123] Sun Q., Lai L., Zhou J., Yi S., Liu X., Guo J., Zheng Y. (2022). Differences in ecological traits between plants grown in situ and ex situ and implications for conservation. Sustainability.

[124] Havens K., Guerrant E.O., Maunder M., Vitt P., Guerrant E.O., Havens K., Maunder M. (2004). Guidelines for ex situ conservation collection management: Minimizing risks. Ex Situ Plant Conservation: Supporting Species Survival in the Wild.

[125] Husband B.C., Campbell L.G., Guerrant E.O., Havens K., Maunder M. (2004). Population responses to novel environments: Implications for ex situ plant conservation. Ex Situ Plant Conservation: Supporting Species Survival in the Wild.

[126] Crutsinger G.M., Collins M.D., Fordyce J.A., Gompert Z., Nice C.C., Sanders N.J. (2006). Plant genotypic diversity predicts community structure and governs an ecosystem process. Science.

[127] Johnson M.T.J., Agrawal A.A. (2005). Plant genotype and environment interact to shape a diverse arthropod community on evening primrose (*Oenothera biennis*). Ecology.

[128] Crawford K.M., Rudgers J.A. (2013). Genetic diversity within a dominant plant outweighs plant species diversity in structuring an arthropod community. Ecology.

[129] Catford J.A., Jansson R., Nilsson C. (2009). Reducing redundancy in invasion ecology by integrating hypotheses into a single theoretical framework. Divers. Distrib..

[130] Wadgymar S.M., Weis A.E. (2017). Phenological mismatch and the effectiveness of assisted gene flow. Conserv. Biol..

[131] Prieto-Benítez S., Morente-López J., Rubio Teso M.L., Lara-Romero C., García-Fernández A., Torres E., Iriondo J.M. (2021). Evaluating assisted gene flow in marginal populations of a high mountain species. Front. Ecol. Evol..

[132] Andersson D.I., Jerlström-Hultqvist J., Näsvall J. (2015). Evolution of new functions de novo and from preexisting genes. Cold Spring Harb. Perspect. Biol..

[133] Kronholm I., Collins S. (2016). Epigenetic mutations can both help and hinder adaptive evolution. Mol. Ecol..

[134] Shan S., Soltis P.S., Soltis D.E., Yang B. (2020). Considerations in adapting CRISPR/Cas9 in nongenetic model plant systems. Appl. Plant Sci..

[135] Jones T.A., Monaco T.A., Larson S.R., Hamerlynck E.P., Crain J.L. (2022). Using genomic selection to develop performance-based restoration plant materials. Int. J. Mol. Sci..

[136] Shamshad M., Sharma A., Çiftçi Y.Ö. (2018). The usage of genomic selection strategy in plant breeding. Next Generation Plant Breeding.

[137] Willi Y., Van Buskirk J., Hoffmann A.A. (2006). Limits to the adaptive potential of small populations. Annu. Rev. Ecol. Evol. Syst..

[138] Huang Y., Tran I., Agrawal A.F. (2016). Does genetic variation maintained by environmental heterogeneity facilitate adaptation to novel selection?. Am. Nat..

